# Spontaneous Postpartum Pneumomediastinum (Hamman’s Syndrome): A Case Report and Review of Chest Pain Management in the Immediate Postpartum Period

**DOI:** 10.7759/cureus.79300

**Published:** 2025-02-19

**Authors:** James S George, Marina Antic, Emilia Petcu, Cristian I Madrid, Igor Dumic, Eric Niendorf, Charles W Nordstrom

**Affiliations:** 1 Hospital Medicine, Mayo Clinic Health System, Eau Claire, USA; 2 Internal Medicine, Mayo Clinic Health System, Eau Claire, USA; 3 Radiology, Mayo Clinic Health System, Eau Claire, USA

**Keywords:** non-cardiac chest pain, postpartum chest pain, postpartum dyspnea, spontaneous pneumomediastinum (spm), vaginal deliver

## Abstract

Spontaneous pneumomediastinum is a rare condition in the postpartum period, characterized by symptoms such as dyspnea, chest pain, subcutaneous neck edema, tachycardia, crepitus, dysphonia, and dysphagia. The Valsalva maneuver, commonly performed during the second stage of vaginal delivery, has been implicated as a key precipitating factor in the pathogenesis of this condition. We report the case of a 25-year-old woman (G1P1001, 39w5d), with a history of smoking, who developed postpartum dyspnea and chest pain 24 hours following an uncomplicated vaginal delivery. A comprehensive diagnostic workup, including a CT scan with intravenous contrast, confirmed severe pneumomediastinum. The patient was managed conservatively with analgesics, supplemental oxygen, and close clinical monitoring. A follow-up chest CT performed 24 hours later demonstrated interval improvement of the pneumomediastinum and an esophagogram excluded the presence of an esophageal tear or rupture. Although spontaneous pneumomediastinum is a rare obstetric complication of normal childbirth, it can present dramatically with chest pain, dyspnea, and hemodynamic instability. Diagnosis is established through a combination of history, clinical presentation, and radiographic findings. Management is conservative and includes analgesics, rest, supplemental oxygen therapy, and bronchodilators. Importantly, other potentially life-threatening causes of postpartum chest pain and dyspnea must be carefully ruled out to ensure timely and appropriate treatment.

## Introduction

Pneumomediastinum (PM) is defined as the presence of free air within the mediastinum [1,2]. Spontaneous pneumomediastinum (SPM) refers to PM that develops in the absence of trauma, intrathoracic medical procedures, or mediastinal injury [2]. The estimated incidence of SPM is 1:44,000 within the general population and is even rarer in pregnancy at 1:100,000 [3]. Although SPM in the postpartum period is typically a benign condition that resolves with conservative management, its presentation can be alarming. Several risk factors have been recognized to increase the probability of developing postpartum spontaneous pneumomediastinum. Primiparity increases the likelihood due to longer labor and greater physical stress. Prolonged labor raises intra-thoracic pressure, as does the intense Valsalva maneuver during delivery. High intra-thoracic pressure, often from difficult labor contributes to alveolar rupture. Pre-existing respiratory conditions like asthma or chronic obstructive pulmonary disease (COPD) heighten vulnerability to pneumomediastinum [1-3]. Symptoms may include dyspnea, chest pain, subcutaneous swelling, dysphonia, dysphagia, vomiting, or hemodynamic instability [1-3]. When evaluating postpartum patients with chest pain or dyspnea, it is critical to exclude other, more severe etiologies that may mimic the presentation of SPM. This case report aims to achieve two objectives: first, to describe this rare condition and compare our patient with those reported in the literature, and second, to outline a systematic approach and differential diagnosis of postpartum patients with chest pain and dyspnea.

## Case presentation

**Figure 1:**
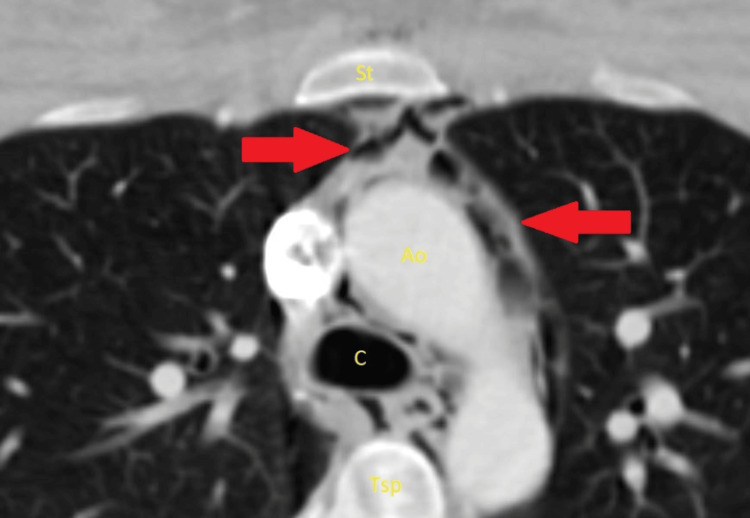
An axial CT image through the upper mediastinum is shown. The arrows indicate foci of gas within the mediastinum, located in retrosternal space and extending around the aorta and carina. St, sternum; Ao, aorta; C, carina; Tsp, thoracic spine.

A 25-year-old primigravida with a past medical history significant only for prior tobacco use experienced an uncomplicated pregnancy, culminating in a spontaneous vaginal delivery. Her labor progressed appropriately without the need for augmentation. Pain management during labor consisted of natural methods, hydrotherapy, and intravenous (IV) fentanyl. Fetal heart rate monitoring during active labor remained category 1 throughout. The patient delivered a viable male infant weighing 3.3 kg, with Apgar scores of 8 and 9. Following delivery she required repair of 1st-degree perineal and right periurethral lacerations. At 24 hours postpartum, the patient developed midsternal chest pain, described as a tight, heavy sensation. The pain was non-radiating and worsened with ambulation. The patient also reported exertional dyspnea, which was absent at rest. There was no associated history of palpitations, lightheadedness, or dizziness, either at rest or with activity. Notably, the chest pain partially improved following the administration of acetaminophen and Maalox. On initial assessment, the patient was hemodynamically stable, with no evidence of tachycardia. Blood pressure measurements obtained from both arms were 113/72 mmHg and 109/65 mmHg. The physical examination revealed an alert and awake patient in no apparent distress. Cardiovascular examination demonstrated normal heart sounds without murmur, gallop, or rub. Respiratory examination revealed nonlabored breathing, with clear lung fields and no wheezes or rales. The abdominal examination was unremarkable and consistent with typical postpartum findings. The lower extremities were warm, well-perfused, and without signs of pitting edema. A broad differential diagnosis was considered, including musculoskeletal chest pain, anxiety, gastroesophageal reflux disease, pulmonary embolism, acute coronary syndrome (ACS), and aortic dissection. Laboratory workup demonstrated normal results for serial troponin levels, complete blood cell count (CBC), and comprehensive metabolic panel (CMP). However, the D-dimer was elevated at 1,964 ng/mL compared to the normal reference range during the third trimester of pregnancy (400-500 ng/mL). An electrocardiogram (ECG) demonstrated sinus rhythm without ischemic changes. Chest X-ray (CXR) was unremarkable. Transthoracic echocardiogram (TTE) revealed a preserved ejection fraction of 62%, with no regional wall motion abnormalities or pericardial effusion. Given the concern for pulmonary embolism, the patient underwent a lower extremity ultrasound, which was negative for acute deep vein thrombosis (DVT). A contrast-enhanced CT scan of the chest was negative for pulmonary embolism but revealed moderately severe pneumomediastinum extending into the lower neck (Figure 1).

To exclude the possibility of esophageal perforation, an esophagogram was performed, which showed no abnormalities. The patient's pneumomediastinum was determined to be spontaneous, with the likely etiology attributed to barotrauma caused by the Valsalva maneuver during vaginal delivery. The patient was managed conservatively with analgesics (acetaminophen and ibuprofen), supplemental oxygen, and close monitoring. A repeat chest CT performed the following day demonstrated a slight improvement in the pneumomediastinum. The patient’s symptoms continued to resolve, and she was discharged in stable condition. At a follow-up appointment with her primary care provider two weeks later, she reported no further complaints and remained in good health.

## Discussion

The pathophysiology of SPM (also known as Hamman’s syndrome) following vaginal delivery is thought to be related to barotrauma. The rupture of marginal alveoli into perivascular tissue planes allows air to become trapped within the mediastinum [1-3]. During the second stage of labor, the Valsalva maneuver generates significant increases in intrathoracic pressure, which, when combined with reduced vascular caliber creates a pressure gradient that facilitates the movement of along broncho-vascular sheaths into the mediastinum. This mechanism has also been observed in other activities associated with abrupt increases in intrathoracic pressure, such as vomiting, screaming, and forceful coughing [1-3].

A recent systematic review of SPM cases associated with vaginal delivery identified the condition as more common in primigravid patients and typically occurring during the second stage of labor [3]. Rarely, as demonstrated in the present case, SPM can occur with a delayed onset, manifesting 24 hours after delivery. In the immediate postpartum period, acute onset of chest pain and dyspnea may be due to benign etiologies; however, it may also portend a life-threatening condition. The critical role of the hospital medicine consultant is to promptly differentiate between benign and potentially fatal etiologies, initiate the appropriate interventions, and provide reassurance in cases where life-threatening conditions have been excluded.

Below we outline the most critical differential diagnoses to consider when evaluating postpartum patients presenting with chest pain and dyspnea.

Thromboembolic disease

Venous thromboembolism (VTE), which encompasses deep venous thrombosis (DVT) and pulmonary embolism (PE), is a critical consideration in postpartum patients with chest pain or dyspnea. In the postpartum period, PE may result from thromboembolism or, less commonly, amniotic fluid embolization. The prothrombotic state of pregnancy and the postpartum period (defined as the six weeks post-delivery) has been well-established, driven by physiological, anatomic, and immunologic changes inherent to pregnancy [4]. In addition to the hypercoagulable state, both preexisting and pregnancy-specific risk factors contribute to the heightened risk of VTE during this time [4,5]. Preexisting risk factors mirror those encountered in the general population, such as obesity, smoking, and inherited thrombophilia. Pregnancy-specific risk factors include primiparity, gestational diabetes, conception via in vitro fertilization (with higher risk in multiple gestations), cesarean delivery (especially if emergent), pre-eclampsia, and infections such as urinary tract infections or intrapartum chorioamnionitis [4]. The risk of VTE varies across gestational periods, being approximately two-fold higher during the first and second trimesters, nine-fold higher during the third trimester, and increasing dramatically to 22-60-fold higher during the postpartum period, particularly within the first three weeks postpartum [4,5]. Despite this significant relative risk, VTE in the postpartum patient remains 10-50 times lower when compared to inpatients at high risk (1-5%) for VTE and more than 10 times lower than patients following hip or knee replacement. However, the number of postpartum VTE cases remains considerable, given the large denominator of over 10 million annual deliveries in Europe and North America [5].

The diagnosis of VTE, particularly PE, can be challenging during pregnancy and the postpartum period. The classic signs and symptoms of VTE include dyspnea, chest pain, cough, tachycardia, tachypnea, unilateral extremity swelling, tenderness, redness, and warmth. However, these clinical manifestations can be confounded by the normal physiologic changes of pregnancy. Additionally, two-thirds of pregnant and postpartum women with PE present with normal oxygen saturation [4]. The commonly used scoring systems for assessing the probability of VTE, such as the Wells or Geneva scores, are not validated in pregnant or postpartum populations, as these groups were excluded from the original studies [4]. Moreover, a D-Dimer level is of limited utility as it increases throughout the pregnancy. Clinicians should consider performing a lower extremity duplex ultrasonography (DUS) initially. If positive for DVT, anticoagulation can be initiated in the hemodynamically stable patient without further evaluation for PE. However, a negative DUS does not exclude the possibility of PE. In such cases, chest imaging with computed tomography pulmonary angiogram (CTPA) or lung perfusion scintigraphy (V/Q scan) is often required. Given that VTE accounts for approximately 9% of pregnancy-related deaths, clinicians should maintain a low threshold for its consideration and testing in pregnant and postpartum patients [4].

The mainstay of treatment for PE in pregnancy is anticoagulation, with low-molecular-weight heparin (LMWH) and unfractionated heparin (UFH) being the preferred agents. Direct oral anticoagulants (DOACs) are not recommended due to insufficient safety and efficacy data in pregnancy. Thrombolysis and invasive thrombectomy are not well studied in pregnancy and are reserved for life-threatening cases.

Amniotic fluid embolism (AFE) is a rare but catastrophic obstetric emergency that occurs during labor or three of 10 immediately postpartum. Although the pathophysiology is not fully understood, it is hypothesized to involve the entry of fetal cells and other components into the maternal circulation, triggering an anaphylactoid reaction and subsequent maternal collapse. AFE should be suspected in any pregnant patient who suddenly develops hypoxia, hypotension, and disseminated intravascular coagulopathy (DIC) during labor or immediately postpartum.

The diagnosis of AFE is clinical and one of exclusion, as no definitive diagnostic test exists. Treatment focuses on aggressive cardiopulmonary resuscitation and management of coagulopathy. According to data from the original national registry, 87% of patients with AFE experienced cardiac arrest, with 40% of these arrests occurring within five minutes of symptom onset [6].

Acute coronary syndrome

Although the risk of ischemic heart disease among young women is low, it remains a leading cause of maternal morbidity and mortality and is associated with significant neonatal complications. The estimated incidence is 6.2 per 100,000 deliveries in high-income countries [7,8]. Maternal mortality rates in these cases are substantial, ranging between 5.1-11% [7-9].

The risk and incidence of cardiovascular events increase due to the physiologic changes of pregnancy, including hypercoagulability and hyperdynamic circulation. This risk is further elevated among women with advanced maternal age, multiparity, African ethnicity, or comorbid conditions such as hypertension, hyperlipidemia, diabetes, smoking, alcohol consumption, or illicit drug use [7,9]. Over the past two decades, the incidence of acute coronary syndrome (ACS) in pregnancy has risen, attributed to increasing maternal age and a growing prevalence of cardiovascular risk factors within the pregnant population [8]. ACS can occur during any stage of the pregnancy, but the highest incidence is observed during the third trimester and in the postpartum period [7,8]. Similar to the general population, pregnant patients with ACS typically present with chest pain and angina. However, delays in presentation and evaluation are common in this population [7]. During labor and delivery, uterine contractions can elevate creatine kinase and myoglobin levels, limiting the diagnostic utility of these biomarkers. High-sensitivity troponin is a more specific biomarker for myocardial injury; however, conditions such as pre-eclampsia and hypertension may also elevate troponin levels further complicating evaluation and management [9].

It is important to recognize that certain ECG variations are considered normal during pregnancy due to physiological changes. These include mild left axis deviation, small non-pathologic Q wave in the inferior and anterolateral leads, T wave inversions in anterior leads and lead III, and sinus tachycardia with supraventricular and ventricular ectopic beats [8]. Among pregnant and postpartum patients with ACS, 75% present with ST-segment elevation myocardial infarction (STEMI), and 25% present with non-STEMI. The underlying etiologies of ACS in this population differ significantly from those in the general population, with common causes including spontaneous coronary artery dissection (SCAD), thrombosis without atherosclerosis, and coronary artery spasm [8]. The urgency for intervention and the therapeutic recommendations do not differ from those of the general population, with immediate reperfusion being the standard of care. Percutaneous intervention remains a cornerstone of management; however, it poses unique challenges, including technical difficulties and potential fetal harm from ionizing radiation, even at low doses. As such, non-invasive methods of diagnostics are preferred when feasible [8,9].

Medical management of ACS in pregnancy involves beta blockers and aspirin. To minimize the need for prolonged dual antiplatelet therapy, bare metal stents are preferred over drug-eluting stents when percutaneous intervention is performed [7]. Low-dose aspirin is considered safe; however, there is limited or no data on the safety of P2Y12 inhibitors, bivalirudin, and glycoprotein IIb/IIIa inhibitors. Clopidogrel is used only when necessary and for the shortest possible duration. The use of angiotensin-converting enzyme (ACE) inhibitors and angiotensin receptor blockers (ARBs) in pregnancy is contraindicated due to their teratogenic potential [7].

Spontaneous coronary artery dissection

While rare in the general population, SCAD is the most frequent cause of ACS in pregnancy, accounting for up to 40% of cases. The incidence of SCAD in pregnancy is estimated at 1.81 per 100,000 pregnancies [10,11]. Maternal mortality associated with pregnancy-related SCAD is 4%, while fetal mortality is reported at 2.5%, based on literature reviews [12]. SCAD is defined as occurring during pregnancy or within three months postpartum, with most postpartum events occurring within the first month, particularly the first two weeks. Pregnancy-associated SCAD is often linked to ST elevation, dysrhythmias, and heart failure with reduced ejection fraction. These presentations are commonly associated with dissection of the left main coronary artery, left anterior descending artery, or multiple coronary vessels, often resulting in larger myocardial infarctions [10,12]. Four of 10 In non-pregnant patients, SCAD is commonly associated with underlying conditions such as fibromuscular dysplasia, autoimmune disorders, and connective tissue disease. In contrast, pregnancy-related SCAD is thought to result from hormonal changes that weaken the coronary artery wall, compounded by increased physiological stress [13,14]. Indirect evidence supports this theory, as case reports have linked SCAD to hormonal changes associated with contraceptive use, in vitro fertilization, and transgender hormone therapy [10-14].

A 2017 retrospective analysis by Havakuk et al. involving 120 patients recommended conservative management and observation for stable patients with SCAD, rather than percutaneous coronary intervention (PCI) [15]. PCI may be considered in cases of hemodynamic instability; however, it is associated with a low success rate and high complication risks, including iatrogenic dissections and propagation of existing dissections. In cases where PCI is unsuccessful, coronary artery bypass (CABG) surgery may be required. Although CABG is associated with improved maternal outcomes, it may negatively impact fetal outcomes. As such, performing a cesarean section prior to surgery should be strongly considered to optimize both maternal and fetal outcomes [15].

GERD and peptic ulcer disease

Gastroesophageal reflux disease (GERD) is a common condition in pregnancy, affecting up to 85% of women [16]. Its etiology is attributed to the physiological changes that compromise the mechanisms preventing gastric contents from refluxing into the esophagus, including hormonal influences and mechanical factors related to the growing uterus. While lifestyle modifications, such as dietary adjustments and positional changes, are often effective in managing symptoms, pharmacologic treatment, such as H2 blockers or proton pump inhibitors, may be required if conservative measures are insufficient. Treatment decisions should consider both maternal and fetal safety. Notably, most women without a prior history of GERD experience symptom resolution following delivery [17].

Peptic ulcer disease (PUD) is less common in pregnancy but can present with symptoms such as nausea, dyspepsia, vomiting, and epigastric pain, particularly at night or after meals. Pregnant women with PUD are more likely to be of Hispanic ethnicity and less likely to have risk factors commonly associated with PUD in the general population, such as smoking, alcohol use, or nonsteroidal anti-inflammatory drug (NSAID) use [18,19]. Studies show that upper GI endoscopy is safe in pregnancy, with no significant complications, labor induction, or fetal malformations observed [18]. However, a retrospective U.S.-based study analyzing data from 2,535 pregnant women matched with over 12,000 non-pregnant controls, found that pregnant women undergoing esophagogastroduodenoscopy (EGD) are at an increased risk of developing VTE [18].

Despite being less likely to undergo diagnostic or therapeutic procedures, such as endoscopy, pregnancy does not appear to worsen outcomes of these interventions [18]. The reasons underlying the reduced utilization of invasive endoscopic procedures, including EGD, remain unclear but are likely multifactorial. Contributing factors may include concerns about maternal and fetal complications associated with anesthesia, technical challenges due to increased intestinal collapsibility in pregnant women, difficulties with positioning to maintain placental blood flow, and the need for fetal monitoring during such procedures, among others.

The decision to perform EGD during pregnancy requires a multidisciplinary approach, with careful consideration of the patient's symptoms, response to medical therapy, and pregnancy-related risks. This evaluation should be conducted on a case-by-case basis by a team that includes an obstetrician, gastroenterologist, primary care provider, and the patient utilizing a shared decision-making process to ensure an individualized and informed approach [19].

Aortic dissection

Chest pain caused by aortic dissection (AD) is an extremely rare occurrence in pregnancy, typically manifesting during the third trimester and puerperium [20,21]. The incidence of AD in pregnancy is reported to be 0.1-0.4% of all cases of aortic dissection and 0.0004% of all pregnancies [19]. The overall mortality rate for aortic dissection in the general population has been reported as high as 60% [20-25], whereas pregnancy-related AD has an inpatient mortality rate of 6.8% [19,25]. The majority of pregnancy-related aortic dissections are type A and tend to occur in the third trimester. In contrast,10-20% are type B dissections, which most commonly arise during the puerperium period [25]. Given that these patients are of reproductive age, the median age of AD in pregnancy is 30.6 years, significantly younger than the median age of 66.8 years observed in the general population [21]. The incidence of aortic dissection increases with advancing maternal age, likely due to the presence of additional risk factors such as hypertension, five of 10 gestational diabetes, and atherosclerosis [21].

The physiologic demands of pregnancy, including increased heart rate, stroke volume, and cardiac output are believed to impose significant stress on an aortic wall, with those effects peaking during the third trimester at 32 weeks of gestation [26-29]. Hormonal changes may further contribute to arterial wall weakening, as histopathological findings in the aortic media during pregnancy have demonstrated a loss of elastic fibers and fragmentation of reticulin fibers [26-29]. Additional risk factors for aortic dissection in pregnancy include underlying conditions such as bicuspid aortic valve disease with aortic root enlargement, Marfan syndrome, Ehlers-Danlos syndrome, and Turner syndrome. In patients with Marfan syndrome, preconception counseling is recommended, with baseline echocardiography to evaluate for aortic dilation, valvular disease, or early signs of aortic dissection. During pregnancy, close monitoring with serial echocardiograms is recommended every four to 12 weeks, and at six months postpartum to ensure timely identification and management of complications.

Pneumothorax

The occurrence of primary spontaneous pneumothorax (PSP) and secondary spontaneous pneumothorax (SSP) during pregnancy is exceedingly rare, with fewer than 100 cases reported in the literature [30,31]. From a management perspective, expert guidelines support conservative measures including aspiration and oxygen supplementation, for most stable women who develop PSP during pregnancy, provided there is no fetal distress [30]. In cases of SSP, pneumothorax occurring during labor and delivery, or when fetal distress is present, chest tube or catheter thoracostomy is indicated [30]. Given the substantial risk of recurrence during future pregnancies, pleurodesis should be offered to women after delivery. For pregnant women with or without a history of pneumothorax who are at risk of developing pneumothorax during labor and delivery, close consultation with thoracic surgery, pulmonary medicine, and obstetrics and gynecology is advised. Most experts advise elective, assisted delivery at or near-term, with regional anesthesia to minimize maternal effort and reduce the risk of pneumothorax recurrence or progression.

Esophageal rupture

Esophageal perforation is a surgical emergency, and patient outcomes are highly dependent on the timeliness of diagnosis and treatment. Mortality is approximately 10% when the condition is diagnosed early and managed promptly; however, this rate rises dramatically to nearly 50% with delayed intervention. While the exact incidence remains unclear, it is estimated to occur at about 3.1 per 1,000,000 individuals annually. The occurrence of esophageal perforation during pregnancy is exceedingly rare, with only a limited number of cases documented in the literature [32].

Esophageal perforation can result from various etiologies, including trauma (both blunt and penetrating), iatrogenic causes (e.g., during difficult endotracheal intubation, esophagogastroduodenoscopy (EGD), or esophageal stenting, the latter being particularly high risk), ingestion of foreign bodies (e.g., dentures in the elderly or fish bones), heavy weightlifting (predominantly in men), and spontaneous rupture, commonly referred to as Boerhaave’s syndrome. In pregnant patients, spontaneous rupture is often exacerbated by physiological changes of pregnancy, including increased intra-abdominal pressure and hormonal effects on the esophageal mucosa [32,33]. Although extremely rare, esophageal perforation can also occur as a complication of childbirth, typically associated with hyperemesis gravidarum [32,33].

The clinical presentation of esophageal perforation typically includes severe retrosternal pain, vomiting, dysphagia, and systemic signs of infection, such as fever and sepsis, which may develop secondary to mediastinitis. Although intense retching and vomiting preceding the onset of pain are frequently reported, approximately 25-45% of patients may not have a preceding history of vomiting [32,33]. About 50% of patients exhibit the classic triad of vomiting, chest pain, and subcutaneous emphysema, collectively referred to as Mackler’s triad [33]. Physical examination may reveal crepitus over the chest wall due to subcutaneous emphysema, respiratory distress, and signs of sepsis and multiorgan failure, typically associated with mediastinitis. In cervical esophageal perforation, symptoms may manifest as neck pain, dysphagia, or dysphonia, accompanied by tenderness over the sternocleidomastoid muscle and crepitus from cervical subcutaneous emphysema. Additionally, neck stiffness, resulting from irritation of the paravertebral fascia due to leaked esophageal contents, is a frequently observed feature in cervical perforations [33].

The diagnosis of esophageal perforation is confirmed through imaging, with contrast-enhanced esophagography or computed tomography (CT) scans regarded as the gold standard. Given the potential for rapid deterioration, prompt and accurate diagnosis is essential. Management strategies are guided by the timing, location, and severity of the perforation, as well as the patient’s clinical status. Small, contained six of 10 perforations may be managed conservatively with parenteral nutrition, broad-spectrum antibiotics, and supportive care. However, larger or more complicated perforations often require surgical intervention. In pregnant patients, maternal and fetal safety must be prioritized when determining treatment strategies, particularly in relation to surgical procedures and imaging modalities. A multidisciplinary approach involving obstetricians, thoracic surgeons, and anesthesiologists is crucial to optimize outcomes for both the mother and fetus.

Acute chest syndrome

Acute chest syndrome should always be considered in the differential diagnosis of chest pain in any pregnant woman with sickle cell disease (SCD) [34-40]. A pooled analysis of studies from low- and middle-income settings estimates maternal mortality in women with SCD to be approximately 2,393 deaths per 100,000 live births with multidisciplinary care and as high as 4,300 deaths per 100,000 live births without such care. In contrast, maternal mortality rates in the general population are significantly lower, at approximately 23.8, and eight deaths per 100,000 live births in the United States and northern Europe, respectively [34-40]. Acute chest syndrome occurs in approximately 50% of patients with SCD, with an all-cause mortality rate of up to 13%. Moreover, recurrence is common, with up to 80% of patients with a history of acute chest syndrome experiencing subsequent episodes [38].

Pregnancy, with its associated physiological changes, increases the risk of acute chest syndrome, particularly during the third trimester and up to one week postpartum [39]. The clinical presentation of acute chest syndrome includes chest pain (84%), fever (64%), shortness of breath (47%), and neurologic findings (22%). The severity of the syndrome can vary widely, ranging from mild symptoms such as chest pain with minimal hypoxia to severe manifestations, including respiratory failure requiring intubation. The diagnosis of acute chest syndrome is based on the presence of a new pulmonary density on chest imaging and at least one of the following: a temperature ≥38.5°C, a decrease in oxygen saturation (SpO2) of >3%, tachypnea, chest pain, cough, wheezing, or rales [37]. Distinguishing acute chest syndrome from pneumonia can be challenging due to overlapping clinical features; however, this should not delay appropriate management. Treatment involves adequate analgesia, broad-spectrum antibiotics, supplemental oxygen, and incentive spirometry. Depending on the severity of the condition, respiratory support may be required, ranging from noninvasive ventilation to mechanical ventilation or, in severe cases, extracorporeal membrane oxygenation.

Musculoskeletal pain

Various musculoskeletal chest wall disorders can cause chest pain during pregnancy, often resulting from physiological changes, hormonal influences, vascular alterations, and mechanical stress exerted by an enlarging uterus, particularly in the third trimester [41-43]. These conditions can lead to significant morbidity and anxiety for the patient, emphasizing the importance of prompt diagnosis and management. Musculoskeletal chest pain can originate from the chest wall, neck, shoulders, diaphragm, and structures below the diaphragm [41,42]. Common causes of musculoskeletal chest pain include costochondritis, rib fractures (stress or traumatic), muscle strain, myofascial pain and fibromyalgia, herpes zoster, and psychogenic regional pain syndrome, among others [41,42]. Diagnosis relies on a detailed patient history, focusing on the location, quality, intensity, duration, and aggravating and alleviating factors, followed by a physical examination. A hallmark feature of musculoskeletal disorders is palpatory tenderness and pain with movement. Importantly, costochondritis can co-occur with other underlying conditions that can cause chest pain; thus, its presence does not exclude alternative or concurrent diagnoses [41]. During the physical exam, it is essential to perform a thorough and systematic examination of the anterior and posterior chest wall for swelling, erythema, heat, and tenderness. Additionally, a neurologic exam should be performed to rule out nerve root compressions originating from cervical and thoracic spinal cord segments. Conservative management is typically recommended for musculoskeletal chest pain during pregnancy. In the general population, NSAIDs and analgesics are the mainstay of therapy. However, during pregnancy, pharmacotherapy (especially NSAIDs) should only be used if the maternal benefit clearly outweighs the potential risk to the fetus. If used, the lowest effective dose for the shortest duration possible should be employed. The use of NSAIDs or high-dose aspirin is not recommended during the third trimester due to the risk of premature closure of the ductus arteriosus and oligohydramnios [44].

Pericarditis

The precise incidence of pericarditis during pregnancy and the postpartum period is not well-defined but it is believed to be similar to the rate observed in the general population [45]. During pregnancy, hypervolemia seven of 10 and hypoalbuminemia contribute to the development of pericardial effusion, which occurs in up to 40% of women during the third trimester and postpartum. These effusions are typically small and asymptomatic [46]. The most common underlying etiology of pericarditis, with or without associated pericardial effusion, is idiopathic, while viral and autoimmune causes account for a minority of cases. Due to the increased blood volume during pregnancy, affected women tend to tolerate pericardial effusions more effectively than their non-pregnant counterparts [45-48].

The clinical presentation of pericarditis during pregnancy is indistinguishable from that in the general population, with patients commonly reporting pleuritic chest pain, chest pain that is alleviated by leaning forward, and dyspnea. However, diagnosing pericarditis in pregnancy can be challenging due to the overlap of symptoms with physiological changes of pregnancy and other potential causes of chest pain and dyspnea. For example, dyspnea and tachycardia are expected physiological changes in late pregnancy, while ECG alterations, such as inverted T waves, are common in this population. Additionally, obtaining TTE images may be challenging, particularly in acquiring subcostal views [46-48]. Additionally, inflammatory markers, including erythrocyte sedimentation rate (ESR) and C-reactive protein (CRP), are often elevated in pregnancy.

Cardiac magnetic resonance imaging (MRI) has limited diagnostic utility for pericarditis during pregnancy due to the contraindication of gadolinium contrast, which poses a risk of fetal renal and cutaneous toxicity [47,48]. However, MRI can be safely employed in the postpartum period, provided maternal renal function is intact. Management strategies for pericarditis in pregnancy and the postpartum period are not extensively addressed in the latest European Society of Cardiology guidelines [49]. During the postpartum phase, including while breastfeeding, the use of aspirin, NSAIDs, colchicine, and prednisone is permissible. In cases where aspirin, NSAIDs, and colchicine fail to manage symptoms, second-line treatment with non-fluorinated corticosteroids, such as prednisone or prednisolone, should be considered. These agents are metabolized more rapidly than fluorinated steroids, minimizing fetal exposure and toxicity [47,48]. A daily dose of prednisone equivalent to 20 mg or less is recommended. If steroid therapy remains ineffective, interleukin-1 (IL-1) inhibitors may be considered as a third-line option. However, data regarding the safety of IL-1 inhibitors in pregnancy and breastfeeding is limited to case reports and small case series, with no definitive evidence supporting their use in these populations [47-49].

Peripartum cardiomyopathy

Peripartum cardiomyopathy (PPCM) is a form of acute heart failure characterized by systolic dysfunction with an ejection fraction of less than 45%. It presents clinically as heart failure that develops during the last month of pregnancy or within the first five months postpartum, in the absence of preexisting cardiac disease [50]. Most cases occur in the postpartum period, most commonly within the first week after delivery [51]. 

The global incidence of peripartum cardiomyopathy is one in 2,000 births, though significant geographic variation exists. The highest incidence is reported in Nigeria, where PPCM affects one in 100 births. In the United States, Black women have a four-fold higher risk of developing PPCM compared to White women. Identified risk factors include pre-existing hypertension, particularly pre-eclampsia, advanced maternal age, untreated anemia, and multiple gestations [51-53]. 

Approximately 60% of cardiogenic shock cases during the peripartum period are caused by peripartum cardiomyopathy, with mortality rates reaching as high as 20%. Mortality is particularly elevated among Black women in the United States and in women from less-developed countries, highlighting racial and geographic disparities in outcomes [50-53]. 

Peripartum cardiomyopathy typically presents with chest pain, dyspnea, hypoxia, or palpitations secondary to arrhythmias. The clinical presentation can mimic other postpartum conditions such as pulmonary embolism, SCAD, and ACS, necessitating a high index of suspicion. Diagnosis is primarily confirmed by echocardiography, which reveals decreased systolic function. Additional diagnostic findings, such as interstitial pulmonary edema on chest X-ray and elevated pro-BNP levels, can further support the diagnosis [50-53].

The management of peripartum cardiomyopathy follows similar principles to the treatment of non-ischemic dilated cardiomyopathy and other forms of heart failure with reduced ejection fraction. Diuretics and nitrates are helpful in alleviating fluid overload; however, their use may lead to hypotension, which is particularly concerning in the peripartum period. Angiotensin-converting enzyme (ACE) inhibitors, angiotensin II receptor blockers (ARBs), and aldosterone receptor antagonists are contraindicated during pregnancy but may be used postpartum. Hydralazine is considered safe for afterload reduction in both pregnancy and the postpartum period. Additionally, beta-blockers are well-tolerated and recommended for use during pregnancy [50-54]. 

The safety of novel heart failure therapies, including sacubitril-valsartan and sodium-glucose cotransporter 2 (SGLT2) inhibitors, during pregnancy and the postpartum period remains unknown due to a lack of clinical data. However, these agents are increasingly utilized in the postpartum management of PPCM. Their safety during breastfeeding has not been well established and requires further investigation [50-54].

## Conclusions

Pneumomediastinum is a rare but notable obstetric complication that can occur during normal vaginal delivery. Clinical evidence suggests that this condition can be effectively managed with conservative measures, including analgesia, rest, and supplemental oxygen. The diagnosis is typically established through clinical evaluation supported by confirmatory imaging, such as chest radiographs or CT scans. This report highlights the rare yet critical association between labor and life-threatening etiologies of chest pain, such as pneumomediastinum, emphasizing the importance of a comprehensive differential diagnosis. This report underscores the necessity of systematically ruling out other potentially fatal conditions and advocates for a thorough diagnostic workup that prioritizes both maternal and fetal safety. A cautious, multidisciplinary approach facilitates the timely identification and appropriate management of this uncommon yet clinically significant obstetric complication.
